# The Early Terrestrial Fungal Lineage of *Conidiobolus*—Transition from Saprotroph to Parasitic Lifestyle

**DOI:** 10.3390/jof8080789

**Published:** 2022-07-28

**Authors:** Andrii P. Gryganskyi, Yong Nie, Ann E. Hajek, Kathie T. Hodge, Xiao-Yong Liu, Kelsey Aadland, Kerstin Voigt, Iryna M. Anishchenko, Vira B. Kutovenko, Liudmyla Kava, Antonina Vuek, Rytas Vilgalys, Bo Huang, Jason E. Stajich

**Affiliations:** 1 UES Inc., Dayton, OH 45432, USA; 2 School of Civil Engineering and Architecture, Anhui University of Technology, Ma’anshan 243002, China; nieyong@ahut.edu.cn; 3 Department of Entomology, Cornell University, Ithaca, NY 14853, USA; aeh4@cornell.edu; 4 Section of Plant Pathology & Plant-Microbe Biology, School of Integrative Plant Science, Cornell University, Ithaca, NY 14853, USA; kh11@cornell.edu; 5 College of Life Sciences, Shandong Normal University, Jinan 250014, China; liuxiaoyong@im.ac.cn; 6 Department of Microbiology and Plant Pathology, University of California, Riverside, CA 92521, USA; kelseya@ucr.edu (K.A.); jason.stajich@ucr.edu (J.E.S.); 7 Department of Molecular and Applied Microbiology, Leibniz Institute for Natural Product Research and Infection Biology, 07745 Jena, Germany; kerstin.voigt@hki-jena.de; 8 M.G. Kholodny Institute of Botany, National Academy of Sciences of Ukraine, 02000 Kyiv, Ukraine; ira_anishchenko@hotmail.com; 9 Agrobiological Department, National University of Life and Environmental Sciences of Ukraine, 03041 Kyiv, Ukraine; virakutovenko@gmail.com (V.B.K.); kavalyuda@ukr.net (L.K.); antoninavuek@mail.ru (A.V.); 10 Department of Biology, Duke University, Durham, NC 27708, USA; fungi@duke.edu; 11 Anhui Provincial Key Laboratory for Microbial Pest Control, Anhui Agricultural University, Hefei 230036, China

**Keywords:** ballistic conidia, entomopathogenicity, evolution, ancestral state reconstruction

## Abstract

Fungi of the *Conidiobolus* group belong to the family Ancylistaceae (Entomophthorales, Entomophthoromycotina, Zoopagomycota) and include over 70 predominantly saprotrophic species in four similar and closely related genera, that were separated phylogenetically recently. Entomopathogenic fungi of the genus *Batkoa* are very close morphologically to the *Conidiobolus* species. Their thalli share similar morphology, and they produce ballistic conidia like closely related entomopathogenic Entomophthoraceae. Ballistic conidia are traditionally considered as an efficient tool in the pathogenic process and an important adaptation to the parasitic lifestyle. Our study aims to reconstruct the phylogeny of this fungal group using molecular and genomic data, ancestral lifestyle and morphological features of the conidiobolus-like group and the direction of their evolution. Based on phylogenetic analysis, some species previously in the family Conidiobolaceae are placed in the new families Capillidiaceae and Neoconidiobolaceae, which each include one genus, and the Conidiobolaceae now includes three genera. Intermediate between the conidiobolus-like groups and Entomophthoraceae, species in the distinct *Batkoa* clade now belong in the family Batkoaceae. Parasitism evolved several times in the *Conidiobolus* group and Ancestral State Reconstruction suggests that the evolution of ballistic conidia preceded the evolution of the parasitic lifestyle.

## 1. Introduction

The subphylum Entomophthoromycotina Humber is comprised of over 300 species which occupy various ecological niches, from saprotrophs to pathogens of insects [1]. Furthermore, some species parasitize a wide range of other hosts from different kingdoms of life: mushroom fruit bodies, fern gametophytes, protists, nematodes, millipedes, spiders, reptiles, and other animals, including humans [2]. Most of the entomopathogenic species are highly specialized to their hosts, and the efficiency of the infection even in closely related arthropod species is very low [3]. However, there are several species of pathogen-generalists that are able to infect the hosts from a wide range of insect families, such as *Batkoa major* [4]. The hallmarks of most fungi of this group are ballistic conidia, which are dispersed from the cadavers of deceased insects and infect new victims. Some of the entomophthoralean fungi can efficiently control the populations of harmful insects in natural and agricultural ecosystems and are thought to be useful as biocontrol agents [5]. 

Species of *Conidiobolus* sensu lato are saprobic or pathogenic fungi with forcibly discharged globose conidia, simple phototropic conidiophores, and secondary conidia similar to the primary, of a different shape, or absent. They have been considered a basal group in the subphylum Entomophthoromycotina. It is clear that their simple morphology has masked their phylogenetic diversity. Humber [6] moved several species to *Batkoa* based on their micromorphology and karyology. Recently, Nie et al. [7] reduced *Conidiobolus* sensu lato from 80 to 37 species and assigned the remaining species to three newly described genera: *Capillidium* B. Huang & Y. Nie, *Microconidiobolus* B. Huang & Y. Nie, and *Neoconidiobolus* B. Huang & Y. Nie. Cai et al. [8] described an allied genus *Azygosporus* B. Huang & Y. Nie as a close relative of *Conidiobolus* sensu stricto. This group of morphologically similar fungi belonging to *Conidiobolus* sensu lato may exceed one hundred species, and discovery of new species continues [9]. 

Most conidiobolus-like fungi are saprotrophs that dwell in organic detritus, and therefore grow well on artificial media under laboratory conditions. They are ubiquitous soil inhabitants, are easy to isolate from a variety of different soils [10], and are common in fungal culture collections across the world. Some of the species have been recorded infecting various insect species, and these are often known as having broad host ranges (ARSEF, ATCC and CBS web pages). Two species are nematode parasites, five can infect other fungi, lichens, and mosses, and nearly a dozen are found in or on dead or living arthropods, mostly insects (Table S1). Some *Conidiobolus* species can infect warm-blooded animals, including humans, and these animal diseases are called conidiobolomycoses [11,12]. Together with morphologically similar species of *Batkoa*, which include insect pathogens of wide and narrow host ranges, species of *Conidiobolus* sensu lato might serve as good examples of the evolutionary transition from a saprotrophic lifestyle to highly specialized parasitism on arthropods, as exhibited in the family Entomophthoraceae which originated from conidiobolus-like ancestors. We see the evolutionary trajectory of entomophthoralean fungi as follows: organic detritus, litter, and soil –> dead insects –> living insects: wide host range, weak pathogens -> living insects: narrow range, strong pathogens [8,9].

**Figure 1:**
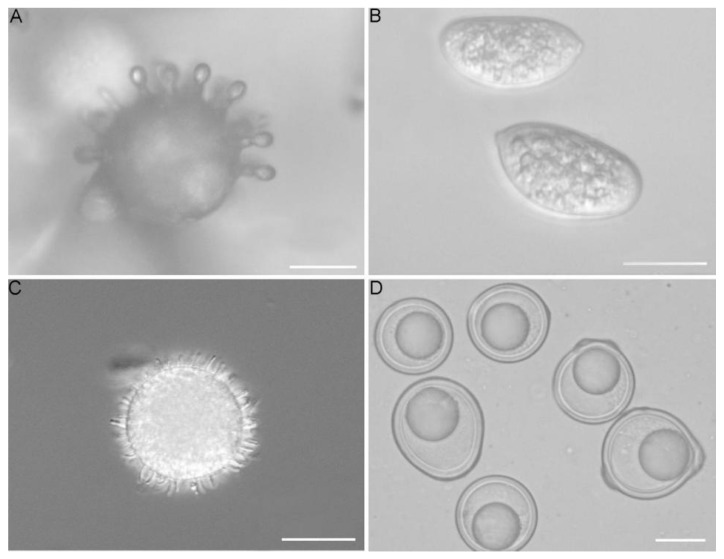
(**A**). *Conidiobolus coronatus* (microconidia); (**B**). *Capillidium macrocapilliconidium* (capilliconidia) [13]; (**C**). *Conidiobolus coronatus* (villose conidia); (**D**). *Conidiobolus brefeldianus* (zygospores); Scale bars: (**A**–**D**) = 20 μm.

Entomophthoralean fungi might acquire this ability to parasitize insects through the process of their evolution as primarily soil inhabitants, first growing on insect cadavers in litter and soil and later infecting living insects. Infecting nematodes, other fungi and lichens, and plants might evolve as secondary adaptations, and therefore are not major evolutionary paths. Sporulation of this fungal group plays an essential role in dissemination and pathogenicity processes. It is diverse with the major spore type being ballistic conidia, with several ejection types (Figure 1).

The goal of our study was to describe the lifestyle of the ancestors of conidiobolus-like fungi, and thus the econiche occupied by the ancestors of Entomophthoromycotina. Our hypothesis is that these ancestors were soil saprotrophs, which over evolutionary time acquired the ability to use dead and later living insects as the substrate for their growth, development, and dissemination. A second goal of our study was to evaluate whether a hallmark feature of Entomophthoromycotina, forcibly discharged conidia, was an adaptation to a parasitic lifestyle, or whether it originated earlier evolutionarily. 

We also removed species of *Conidiobolus* sensu lato from the family Ancylistaceae which has an atypical type genus *Ancylistes*—pathogens of algae. Relationships of this genus will be possible to resolve based on genome sequences of its species (T. James, personal communication). Based on the data obtained through the phylogenetic reconstruction, we elevated the ranks of the genera *Batkoa*, *Capillidium*, *Conidiobolus* sensu stricto, and *Neoconidiobolus* to the family level. Our study aims to organize these morphologically similar yet genetically diverse fungal groups based on revealing their evolutionary trajectories. 

## 2. Materials and Methods

### 2.1. Phylogenetic Reconstruction

We used molecular data (single copy genes and 17 available genomic/transcriptomic datasets) of 41 species of the genera *Batkoa*, *Capillidium*, *Conidiobolus*, *Microconidiobolus*, and *Neoconidiobolus* in our study. All of these species morphologically resemble *Conidiobolus*, and often have similar lifestyles. We considered the size of the genera as small if they had less than five taxa, and large for the genera with more than ten species. We also included the molecular data of species in the family Entomophthoraceae, including eight genomic and transcriptomic datasets. As outgroups we used sequences of *Schizangiella serpentis* and genomic data of *Syncephalis pseudoplumigaleata* S71, *Smittium culicis* ARSEF 9010, and *Coemansia reversa* NRRL 1564 [14].

The alignment with proteins and DNA was combined with partitions in NieGenomeComb.mfa. The partitions file is from Nie et al. [7] NieGenomeComb.partitions.nex and the IQTREE best merged partitions were used to create combined alignment NieGenomeComb.partitions.nex.best_scheme.nex, submitted to TreeBase (S29885).

Phylogenetic reconstruction was done by combining single-gene trees [7], which were rebuilt using a partitioned subset of overlapping taxa, and genome-scale coalescent-based species tree estimation using ASTRAL [15] to compare the multipartitioned concatenated topology. The resulting combined coalescent trees contained 400 protein-coding genes. Trees were tested for congruence and compared to the tree built with just genome and transcriptome data. A bootstrap analysis was done using 1000 repetitions. The phylogenetic tree was visualized using FigTree v 1.4.4 [16], saved as a PDF file and adjusted in Microsoft Office PowerPoint.

### 2.2. Ancestral States Reconstruction (ACR)

Maximum likelihood ancestral character states were reconstructed in R [17] using the rerooting method in phytools [18] under the equal rates, single parameter ER model. Two-character states were reconstructed—lifestyle and ballistic conidia, with each having four conditions. Conditions for lifestyle (only evaluating entomopathogenicity) were (0) non-pathogen, (1) pathogen of wide host range, (2) pathogen of moderate range, and (3) pathogen of narrow range. Conditions for ballistic conidia were (0) not forcible or absent, (1) forcible small and round (≤20 µm), (2) forcible large and round (>20 µm), and (3) forcible other than round. Additionally, ASR was performed using Mesquite [19] for the same two characters. Conditions for lifestyle (only evaluating entomopathogenicity) were (0) non-pathogen and (1) pathogen. Conditions for ballistic conidia were (0) not forcible or absent, (1) forcible. For each ancestral node on both the lifestyle and ballistic conidia trees, the log-likelihood was plotted as a pie graph of colors corresponding to the two or four conditions for each character.

### 2.3. Light Microscopy

Microscopic structures were observed under a BX51 light microscope and imaged with a DP25 microscope-camera system (Olympus Corporation, Tokyo, Japan) to obtain the photoplates. Images used for figures were processed with Adobe Photoshop CS3 Extended version 10.0 software (Adobe Systems, San Jose, CA, USA).

## 3. Results

### 3.1. Phylogenetic Reconstruction

**Figure 2:**
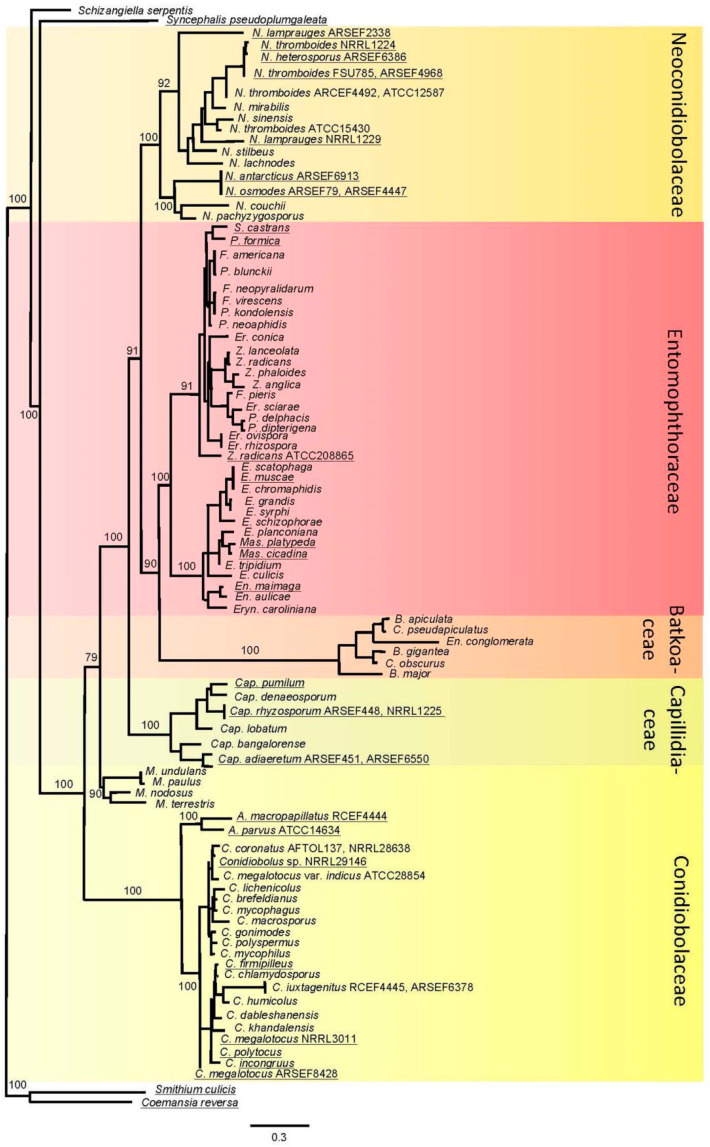
Maximum likelihood phylogenetic tree of conidiobolus-like fungi and related groups. Bootstrap values shown only at the basal nodes. In the genus *Batkoa,* the old taxonomic names of *B. apiculata* (*Conidiobolus pseudapiculatus* and *Entomophaga conglomerata*) and *B. gigantea* (*C. obscurus*) are shown to demonstrate the difficulties of species identification using only morphological features. Underlined are the taxa with existing NGS data. Strain names are shown for the taxa where more than one strain was used. Background colors are various shades of yellow for the mostly saprotrophic families of *Conidiobolus*-like fungi, orange for culturable entomopathogenic Batkoaceae, and red for strict entomopathogens of Entomophthoraceae. A.—*Azygosporus*, B.—*Batkoa*, C.—*Conidiobolus*, Cap.—*Capillidium*, E.—*Entomophthora*, En.—*Entomophaga*, Er.—*Erynia*, Eryn.—*Eryniopsis*, F.—*Furia*, M.—*Microconidiobolus*, Mas.—*Massospora*, N.—*Neoconodiobolus*, P.—*Pandora*, S.—*Strongwellsea*, Z.—*Zoophthora*.

Phylogenetic reconstruction based on the combined 4-gene (18S, 28S, EF-1α and mtSSU) and genome dataset revealed the polyphyletic composition of *Conidiobolus* sensu lato (Figure 2). It consists of several large clades, and is well supported, with bootstrap values between 90 and 100. This topology is also preserved when only genome data were used (Figure S1). 

For most clades we have assigned new families. Basal to all sampled Entomophthoromycotina is a new family Conidiobolaceae fam. nov., described below. This is the group with the most molecular data: both single genes and genome data are available for 17 species. Adjacent and intermediate to it is a small group well supported by bootstrap values which includes representatives of the genus *Microconidiobolus*. It is represented only by single gene data and therefore it would be meaningful to collect more data to determine the affinities of this genus. Another big new family, Capillidiaceae fam. nov., consists of ten species, as shown below. 

Another large group of *Conibiobolus* sensu lato, represented by 18 sets of molecular data for 11 species forms the Neoconidiobolaceae fam. nov., which in our phylogenetic reconstruction is located next to the entomopathogenic species of two families: Batkoaceae fam. nov., and Entomophthoraceae.

The Batkoaceae fam. nov. arises basal to the entomopathogenic Entomophthoraceae, on a branch that is well separated with strong bootstrap support. It includes several *Conidiobolus*-like taxa *C. obscurus* (=*Batkoa gigantea* or *B. obscura*), *C. pseudapiculatus*, and *Entomophaga conglomerata* (=*B. apiculata*).

### 3.2. Taxonomy

#### 3.2.1. **Batkoaceae** Gryganskyi, A.E. Hajek & Stajich, fam. nov. [MB 844345] 

*Type genus:Batkoa* Humber, Mycotaxon 34 (2): 446 (1989) (Humber 1989), [MB 25280].

*Type species:Batkoa apiculata* (Thaxter) Humber, Mycotaxon 34 (2): 446 (1989), [MB 135576].

=*Entomophthora apiculata* (Thaxt.) M.A. Gust., Kungliga Landbruks-Höngskolans Annaler 31: 131 (1965) [MB 330591].

=*Conidiobolus apiculatus* (Thaxt.) Remaud. & S. Keller Mycotaxon 11 (1): 330 (1980) [MB 118560].

*Description*: Mycelia of hyaline, septate, branching hyphae. Hyphal bodies hypha- or ameboid-like, subglobose to elongate, multinucleate, nuclei staining with aceto-orcein. Primary conidiophores simple, positively phototropic, bearing a single apical primary conidium. Primary conidia forcibly discharged, single-celled, multinucleate, globose, with prominent conical papilla. Replicative conidia similar and smaller than primary conidia. Chlamydospores globose, hyaline. Zygospores globose, hyaline or yellowish. Rhizoids present or absent. Obligate pathogens of insects.

*Notes:* Members of the Batkoaceae are entomopathogenic, infecting insects from various orders, ballistospore-forming fungi with a broad global distribution. Their cultures are easily to isolate on simple culture media. 

*Accepted species:*

*Batkoa amrascae* S. Keller & Villac., Philippine Entomologist 11 (1): 81 (1997) [MB 313160].

*Batkoa apiculata* (Thaxt.) Humber, Mycotaxon 34 (2): 446 (1989) [MB 135576].

*Batkoa cercopidis* (S. Keller) B. Huang, Humber & K.T. Hodge, Mycotaxon 100: 231 (2007) [MB 510686].

*Batkoa dysderci* (Viegas) Humber, Mycotaxon 34 (2): 446 (1989) [MB 135577].

*Batkoa gigantea* (S. Keller) Humber, ibid. [MB 135578].

*Batkoa hydrophila* S. Keller, Sydowia 59 (1): 77 (2007) [MB 529508].

*Batkoa limoniae* (S. Keller) S. Keller, Nova Hedwigia 73 (1–2): 171 (2001) [MB 484564]

*Batkoa major* (Thaxter) Humber, Mycotaxon 34 (2): 446 (1989) [MB 135579].

***Batkoa obscura*** (Hall & Dunn) Gryganskyi, comb. nov. [MB 844349].

*Basionym: Entomophthora obscura* I.M. Hall & P.H. Dunn, Hilgardia 27: 162 (1957) [MB 297265] = *Conidiobolus obscurus* (I.M. Hall & P.H. Dunn) Remaud.; S. Keller, Mycotaxon 11 (1): 331 (1980) [MB 118567].

*Batkoa papillata* (Thaxter) Humber, Mycotaxon 34 (2): 446 (1989) [MB 135580].

*Batkoa pseudapiculata* (S. Keller) B. Huang, Humber & K.T. Hodge, Mycotaxon 100: 231 (2007) [MB 510687].

#### 3.2.2. **Capillidiaceae** Y. Nie, Stajich & K.T. Hodge, fam. nov. [MB 844346]

*Type genus: Capillidium* B. Huang & Y. Nie, MycoKeys 66: 62 (2020) [2] [MB 831596]

*Type species: Capillidium heterosporum* (Drechsler) B. Huang & Y. Nie, MycoKeys 66: 62 (2020) [MB 831601] = *Conidiobolus heterosporus* Drechsler, Am. J. Botany 40: 107 (1953) [MB 295472]

*Description:* Mycelia hyaline. Primary conidiophores simple, bearing single primary conidia. Primary conidia forcibly discharged, multinucleate, hyaline, globose, pyriform to obovoid. Two kinds of replicative conidia, the first similar and smaller than primary conidia, the second (capilliconidia) arise singly, off-axis at the top of slender, elongate conidiophores, and are not forcibly discharged. Zygospores present or absent, formed in axial alignment with conjugating segments, globose to subglobose, often smooth, sometimes rough, hyaline or yellowish.

*Accepted species:*

*Capillidium adiaeretum* (Drechsler) B. Huang & Y. Nie, MycoKeys 66: 61 (2020) [MB 831602]

*Capillidium bangalorense* (Sriniv. & Thirum.) B. Huang & Y. Nie, ibid. [MB 831607]

*Capillidium denaeosporum* (Drechsler) B. Huang & Y. Nie, ibid. [MB 831608]

*Capillidium heterosporum* (Drechsler) B. Huang & Y. Nie, ibid. [MB 831601]

*Capillidium jiangsuense* B. Huang & Y. Nie, MycoKeys 89: 146 (2022) [MB 842228]

*Capillidium lobatum* (Sriniv. & Thirum.) B. Huang & Y. Nie, MycoKeys 66: 62 (2020) [MB 831609]

*Capillidium macrocapilliconidium* B. Huang & Y. Nie, MycoKeys 89: 143 (2022) [MB 842227]

*Capillidium pumilum* (Drechsler) B. Huang & Y. Nie, MycoKeys 66: 61 (2020) [MB 831610]

*Capillidium rhysosporum* (Drechsler) B. Huang & Y. Nie, ibid. [MB 831611] 

*Capillidium rugosum* (Drechsler) B. Huang & Y. Nie, ibid. [MB 842229].

#### 3.2.3. **Conidiobolaceae** B. Huang, Stajich & K.T. Hodge, fam. nov. [MB 844347]

*Type genus: Conidiobolus* Bref., Untersuchungen aus dem Gesamtgebiete der Mykologie 4: 35 (1884) [20] [MB 20144]

*Type species: Conidiobolus utriculosus* Bref., Untersuchungen aus dem Gesamtgebiete der Mykologie 4: 35 (1884) [MB 144259]

*Description:* Mycelium of hyaline, septate, branching hyphae. Hyphae multinucleate, with small nuclei that do not stain with aceto-orcein. Simple hyphal conidiogenous cells each develop one apical conidium, a ballistospore that is forcibly discharged by rapid circumcissile rupture and papillar eversion. Detached conidia are hyaline, single-celled, more or less globose, with an everted blunt conical papilla where they were once attached to the conidiophore. Sexual zygospores, present or absent, formed in axial alignment with conjugating segments, globose to subglobose.

*Notes:* Members of the Conidiobolaceae are saprobic, ballistospore-forming fungi with a broad global distribution. They grow readily on simple culture media, typically as white to cream colonies, often forming satellite colonies derived from the asexual ballistospores. The primary ballistospores may germinate in three ways: to form a smaller ballistospore of similar form as the parent spore, to form a passively dispersed capilliconidium atop a long, attenuated stalk, or they may form a hyphal germ tube. Some species are able to opportunistically infect humans and other animals.

**Conidiobolaceae** B. Huang, Stajich & K.T. Hodge

**Included genera (3) and species:**

***Azygosporus*** B. Huang & Y. Nie, MycoKeys 85: 165 (2021) [MB 840849]

*Accepted species:*

*Azygosporus macropappilatus* B. Huang & Y. Nie, ibid. [MB 840848]

*Azygosporus parvus* (Drechsler) B. Huang & Y. Nie, ibid. [MB 840850]

***Conidiobolus*** sensu stricto according to B. Huang & Y. Nie 2020 [MB 20144]

*Accepted species:*

*Conidiobolus coronatus* (Costantin) Batko, Entomophaga 2: 129 (1964) [MB 283037]

*Conidiobolus bifurcatus* B. Huang & Y. Nie, MycoKeys 73: 137 (2021) [MB 831599]

*Conidiobolus brefeldianus* Couch, American Journal of Botany 26: 119 (1939) [MB 258852]

*Conidiobolus dabieshanensis* Y. Nie & B. Huang, Mycosphere 8 (7): 811 (2017) [MB 552756]

*Conidiobolus gonimodes* Drechsler, Mycologia 53: 292 (1961) [MB 328751]

*Conidiobolus iuxtagenitus* S.D. Waters & Callaghan, Mycological Research 93 (2): 223 (1989) [MB 135617]

*Conidiobolus khandalensis* Sriniv. & Thirum., Mycologia 54 (6): 692 (1963) [MB 328754]

*Conidiobolus lichenicola* Sriniv. & Thirum., Mycopathologia et Mycologia Applicata 36: 344 (1968) [MB 328755]

*Conidiobolus lunulus* D. Goffre, R.A. Humber & P.J. Folgarait, Mycologia: 131 (1): 56 (2020) [MB 834443] should be in this group by morphology.

*Conidiobolus macrosporus* Sriniv. & Thirum., Mycologia 59: 702 (1967) [MB 328757]

*Conidiobolus mycophagus* Sriniv. & Thirum., Sydowia 19 (1–6): 88 (1965) [MB 328759]

*Conidiobolus mycophilus* Sriniv. & Thirum., ibid. [MB 328760]

*Conidiobolus polyspermus* Drechsler, Mycologia 53: 279 (1961) [MB 328763]

*Conidiobolus polytocus* Drechsler, American Journal of Botany 42: 793 (1955) [MB 295480]

*Conidiobolus taihushanensis* B. Huang & Y. Nie, MycoKeys 73: 140 (2021) [MB 835124]

*Conidiobolusutriculosus* Bref., Untersuchungen aus dem Gesamtgebiete der Mykologie 4: 35 (1884) [MB 144259] HOLOTYPE SPECIES

*Conidiobolus variabilis* B. Huang & Y. Nie, MycoKeys 73: 142 (2021) [MB 835125]

Additional species to be considered: *C. chlamydosporus*, *C. firmipileus*, *C. humicolus, C. incongruous* and *C. megalotocus*.

***Microconidiobolus*** B. Huang & Y. Nie, MycoKeys 66: 72 (2020) [MB 831597]

*Accepted species:*

*Microconidiobolus nodosus* (Sriniv. & Thirum.) B. Huang & Y. Nie, ibid. [MB 831624]

*Microconidiobolus paulus* (Drechsler) B. Huang & Y. Nie, ibid. [MB 831605]

*Microconidiobolus terrestris* (Sriniv. & Thirum.) B. Huang & Y. Nie, ibid. [MB 831625]

Additional species to be considered: *Microconidiobolus undulatus*.

#### 3.2.4. **Neoconidiobolaceae** X.Y. Liu, Stajich & K.T. Hodge, fam. nov. [MB 844348]

*Type genus: Neoconidiobolus* B. Huang & Y. Nie, MycoKeys 66: 70 (2020) [MB 831598]

*Type species:Neoconidiobolus thromboides* (Drechsler) B. Huang & Y. Nie, MycoKeys 66: 70 (2020) [MB 831606] = *Conidiobolus thromboides* Drechsler, J. Washington Acad. Sci. 43: 38 (1953) [MB 295484]

*Description:* Mycelia hyaline. Primary conidiophores are simple or sometimes branched, positively phototropic, bearing a single apical primary conidium. Primary conidia forcibly discharged, multinucleate, hyaline, globose, pyriform to obovoid. Replicative conidia similar and smaller than primary conidia. Chlamydospores globose, formed terminally on hyphae or from globose cells by thickening of the wall. Zygospores formed in axial alignment with two conjugating segments, globose to ellipsoidal, smooth, hyaline, rarely pale yellowish.

*Notes:* Species of the family Neoconidiobolaceae resemble those of the Conidiobolaceae in lacking both microconidia and capilliconidia. All members in the clade of Neoconidiobolus share the following characteristics: forcibly discharged, hyaline, globose, pyriform to obovoid primary conidia. Two kinds of replicative conidia are produced: One is discharged, similar to and smaller than primary conidia; the other is elongate and forcibly discharged. Two types of resting spores are produced: zygospores and chlamydospores.

*Accepted species:*

*Neoconidiobolus couchii* (Sriniv. & Thirum.) B. Huang & Y. Nie, MycoKeys 66: 73 (2020) [MB 831626]

*Neoconidiobolus kunyushanensis* B. Huang & Y. Nie, Mycological Progress 20: 1233 (2021) [MB 831600]

*Neoconidiobolus lachnodes* (Drechsler) B. Huang & Y. Nie, MycoKeys 66: 73 (2020) [MB 831627]

*Neoconidiobolus mirabilis* (Y. Nie & B. Huang) B. Huang & Y. Nie, ibid. [MB 831628]

*Neoconidiobolus osmodes* (Drechsler) B. Huang & Y. Nie, ibid. [MB 831629]

*Neoconidiobolus pachyzygosporus* (Y. Nie & B. Huang) B. Huang & Y. Nie, ibid. [MB 831630]

*Neoconidiobolus sinensis* (Y. Nie, X.Y. Liu & B. Huang) B. Huang & Y. Nie, ibid. [MB 831631]

*Neoconidiobolus stilbeus* (Y. Nie & B. Huang) B. Huang & Y. Nie, ibid. [MB 831632]

*Neoconidiobolus stromoideus* (Sriniv. & Thirum.) B. Huang & Y. Nie, ibid. [MB 831633]

*Neoconidiobolus thromboides* (Drechsler) B. Huang & Y. Nie, ibid. [MB 831606]

*Neoconidiobolus vermicola* (J.S. McCulloch) B. Huang & Y. Nie, ibid. [MB 831634]

## 4. Ancestral State Reconstruction

The ancestors of entomophthoralean fungi were with high probability saprotrophic, as with most of their extant basal lineages *Azygosporus*, *Conidiobolus*, *Capillidium*, *Microconidiobolus* and *Neoconidiobolus*. The ability to infect insects was developed in various groups and multiple times during the evolution of these fungi. Of the 18 species included in the Conidiobolaceae, *C. macrosporus* is host specific pathogen. Of the 11 species included in *Neoconidiobolus,* two are pathogens with broader host ranges (*Neoconidiobolus osmodes* and *Neoconidiobolus thromboides*). None of the species included in Capillidiaceae (three *Microconidiobolus* and six *Capillidium*) are pathogens. Also, ancestors of the entomophthoralean fungi became entomopathogenic and didn’t lose this ability further on; all extant members of this group (families Batkoaceae and Entomophthoraceae) are entomopathogenic (Table S1). 

**Figure 3:**
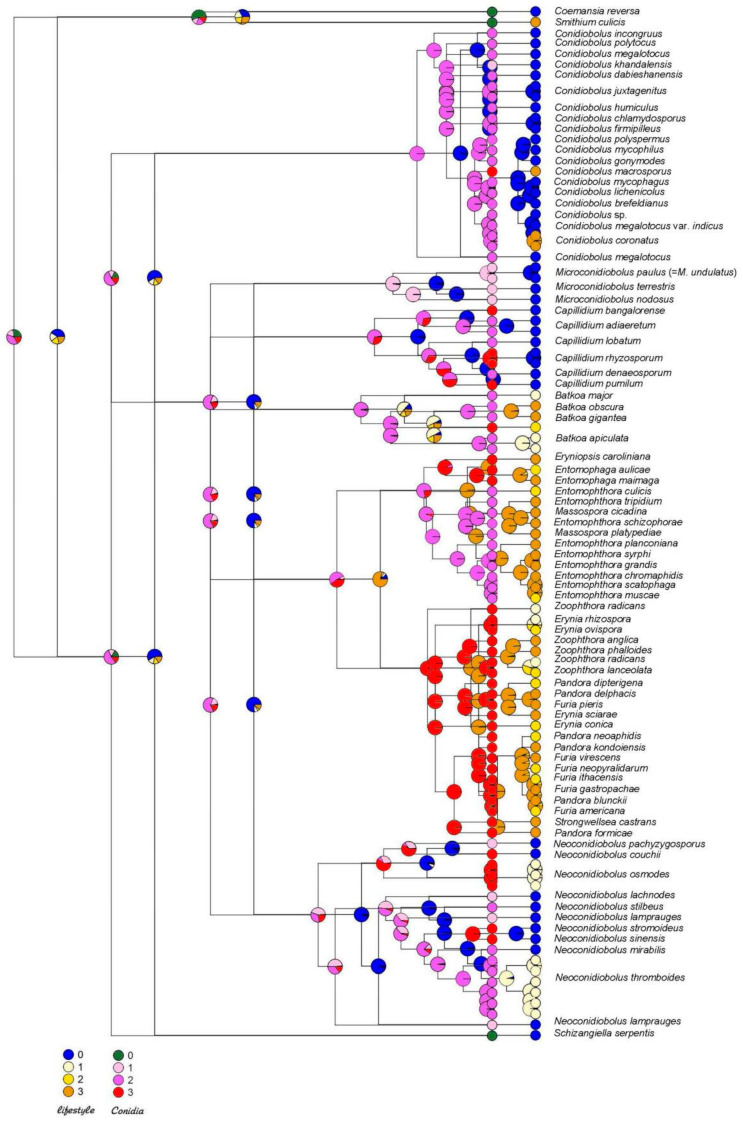
Ancestral State Reconstruction suggests ballistic conidia evolutionarily precedes the parasitic lifestyle. Reconstruction is done for four states. Lifestyle: non-pathogenic to insects, grow well on artificial media (0, blue), insect pathogen of wide range, growing well on artificial media (1, beige), insect pathogen of moderate range (2, yellow), insect pathogen of narrow range (3, orange). Ballistic conidia: not forcible, absent, or unknown (0, green), forcible small and round ≤20 µm (1, pink), forcible large and round (>20 µm) in diameter (2, purple), presence of forcible conidia other than round (3, red).

A similar evolutionary trajectory was reconstructed for another character—ballistic conidia. This character evolved very early in Entomophthoromycotina, in the ancestors with saprotrophic lifestyles, possibly as an adaptation for spore dissemination (Figure 1), and this character is seen throughout the phylogenetic tree for these groups. Ballistic conidia preceded the entomopathogenic lifestyle since this ability is attributed to the earlier (lower) basal nodes on the phylogenetic tree (Figure 3 and Figure S2). This character was inherited by the early entomopathogens and served as an efficient tool for insect infection as taxa evolved further. 

## 5. Discussion

Phylogenetic reconstruction suggests polyphyletic origins of conidiobolus-like fungi, and not a single origin. The polyphyletic origin of this composite fungal group was already suggested by our previous works [7,21,22,23,24], and one of the tasks of this study was to determine the corresponding taxonomic levels of the main branches. Alternatively, the whole group should be treated as a single family (the Entomophthoraceae), and all genera could be subsumed into it. However, we believe that the polyphyletic origin of most branches at the base of Entomophthorales also suggests independent origins of the newly described families.

There are some differences in the placement of certain groups and taxa. In the study of Nie et al. [7], the genera *Conidiobolus* and *Microconidiobolus* were grouped together, while on our tree they are located separately. Therefore, we haven’t assigned a corresponding unique taxonomic level to *Microconidiobolus* that is higher than genus, as we do not yet have genomic or transcriptomic data. Species of *Microconidiobolus* differ morphologically in producing microconidia and this is not characteristic of other members of the Conidiobolaceae. Taxonomic level needs more taxa and more genomes involved for phylogenetic reconstruction.

Also, there are some discrepancies inside the family Entomophthoraceae. The issue with having *Massospora* and two species of *Entomophthora* together in one clade is caused by the lack of the genes for both *Entomophthora* species. Their missing data might cause some interference with better gene sampling for *Massospora*. In any case, *Massospora* is next to *Entomophthora*, as in most previous phylogenetic reconstructions. 

*Neoconidiobolus thromboides* appears in our phylogenetic reconstruction not as a single clade. This might be an indication that this taxon represents a species complex or group of species, and reflects the diversity of this fungal group, which is similar to another complex species, *Conidiobolus coronatus*. The resolution of these two species complexes possibly containing several cryptic species needs better genome sampling. The presence of *Neoconidiobolus heterosporus* inside it can provide a hint. In any case, the spore sizes and especially hosts and substrates of *Neoconidiobolus thromboides* are definitely worth studying in more detail with support from genome references.

The division of Entomophthoromycotina reflects the gradual evolutionary switch from saprotrophy to the parasitic lifestyle, with multiple origins. While all Entomophthoraceae are insect pathogens, conidiobolus-like fungi occupy more diverse ecological niches. They developed the adaptation of infecting insects several times during their evolution. The family Batkoaceae presents intermediate placement between these two groups. While all known species in the Batkoaceae are insect pathogens, they are easy to culture on artificial nutrient media like *Azygosporus*, *Capillidium*, *Micro*-, *Neo*- and *Conidiobolus* isolates. Only a few species of Entomophthoraceae grow well under laboratory conditions in pure culture, mostly requiring special nutritive media, and the majority of species in this fungal lineage are not yet culturable [6]. Typical entomopathogenic fungi of the family Entomophthoraceae have rather atypically large genomes for the Zoopagomycota, being over 600 Mb. Sizes of known genomes from species in the *Conidiobolus* group range from 25 to 90 Mb and this is rather more typical for the saprotrophs and insect symbionts in terrestrial fungal lineages [25].

Similarly to the parasitism of insects, parasitism of other groups of living organisms like lichens, nematodes, and mushrooms developed independently several times in different branches among saprotrophic conidiobolus-like fungi over their evolutionary trajectories. Ancestral state reconstruction for lifestyle and ballistic conidia appearance suggests that these two features coexisted for a long-time during evolution, and ballistic conidia were present in the early ancestors of the whole group already, before pathogenic lifestyles were adopted. Ballistic conidiospores were already present at the very beginning of Entomophthorales evolution in saprotrophic lineages. Although ballistic conidia were described as a hallmark of entomopathogens, they could not be an adaptation to the parasitic lifestyle, namely an adaptation to infect insects. However, the ability of these fungi to eject spores for significant distances, possibly along with light sensing mechanisms, and involving attachment to the substrate due to conidiophore content [26], essentially increased their chances of finding new habitats or infecting hosts, when they had switched to pathogenic lifestyles. Ballistic conidia evolved earlier and was successfully adopted by newly evolved entomopathogens.

Our study aims to add some structure to the very unclear taxonomic positioning of conidiobolus-like fungi. Despite many morphological similarities, they have polyphyletic origins and occupy various econiches ranging from saprotrophy to entomopathogenicity. We hope that our study will help the researchers of this fungal group to assess relatedness between these families and with the entomopathogenic Entomophthoraceae, and also predict the ecological niches of new species of these families, as further discoveries continue.

## Data Availability

All data underlying the presented study will be made available at an online data repository.

## Supplementary Materials

The following supporting information can be downloaded at: https://www.mdpi.com/article/10.3390/jof8080789/s1, Figure S1: Phylogeny based on 27 genomes of Conidiobolus-like fungi; Figure S2: Ancestral state reconstruction of insect-pathogenic lifestyle and ballistic conidia (gray), which evolutionary precedes parasitic lifestyle (black) for two states: present (black) and absent (white); Table S1: Taxa used for phylogenetic and ancestral state reconstructions, their pores and lifestyle.
