# Supplementary material for: The Early Terrestrial Fungal Lineage of Conidiobolus—Transition from Saprotroph to Parasitic Lifestyle
Source: J Fungi (Basel). 2022 Jul 28;8(8):789. doi: 10.3390/jof8080789 (PMC9409958; doi:10.3390/jof8080789)
Supplement: Supplementary file 1 [file jof-08-00789-s001.zip › jof-1778035-supplementary.pdf]

Supplementary Table S1. Taxa used for phylogenetic and ancestral state reconstructions, their pores and lifestyle.

Species new and old name, reference	Lifestyle, range		Primary conidia size in µm	Secondary conidia
	substrate	host range		
**_Capillidium adiaeretum_** = _Conidiobolus adiaeretus_ (Nie et al. 2020)	Organic detritus and plant debris 0		Globose 15-45x13-42 1	Microconidia & capilliconidia 2
**_Capillidium bangalorense_** = _Conidiobolus bangalorensis_ (Balazy 1993)	Soil and litter 0		Subglobose to pyriform 20-25x13-16 2	Like primaries 1
**_Capillidium denaeosporum_** = _Conidiobolus denaeosporus_ (Drechsler 1957, King 1976, Nie et al. 2020, Index Fungorum)	Organic detritus 0		Globose to turbinate 6-21x 13-32 1	Capilliconidia 2
**_Capillidium heterosporum_** = _Conidiobolus heterosporus_ (Nie et al. 2020, ARSEF) [T]	Organic detritus 0		Globose to subglobose 12-37x11-31 1	Like primaries & capilliconidia 2
**_Capillidium lobatum_** = _Conidiobolus lobatus_ (Balazy 1993)	Organic detritus 0		Globose 21-26x20-24 1	-//- 2
**_Capillidium pumilum_** = _Conidiobolus pumilus_ (ibid., ARSEF)	Organic detritus 0		Subglobose to turbinate 9-32x6-25 2	-//- 2
**_Capillidium rhyzosporum_** = _Conidiobolus rhyzosporus_ (ibid.)	Organic detritus 0		Subglobose 22-36x18-30 1	-//- 2
Conidiobolus brefeldianus (Balazy 1993)	Contaminant of fungi 1		Subglobose 21-36x10-31 1	Microconidia 1
Conidiobolus chlamydosporus (Drechsler, 1955c) syn. of _C. firmipilleus_ according to Balazy (1993), Nie et al. (2020)	Organic detritus 0		globose 25 1	-//- 1
**_Conidiobolus coronatus_** (Batko 1964, Balazy 1993, Nie et al. 2020, ARSEF)	Organic detritus & dead & living insects, fungi & warm-blooded animals, wide range 2		Globose 36-52x42-65 1	-//- 1
Conidiobolus dabieshanensis (Nie et al. 2017, Index Fungorum)	Organic detritus 0		Globose to subglobose 33-45x20-38 1	Like primaries 1
**_Conidiobolus firmipilleus_** (Balazy 1993, ARSEF) _C. chlamydosporus_ synonym to this species	Organic detritus & insects, narrow range 3		Subspherical 8-50x7-45 1	Microconidia 1
Conidiobolus gonimodes (Drechsler 1961)	Organic detritus 0		Globose 11-33x12-39 1	-//- 0
**_Conidiobolus incongruus_** (Balazy 1993)	Organic detritus & warm-blooded animals 1		Globose to ellipsoid 12-42x11-37 1	-//- 1
Conidiobolus iuxtagenitus (Nie et al. 2020, Index Fungorum, ARSEF)	Organic detritus & alive insects, narrow range 3		Globose 30-45x16-22 1	Like primaries & microconidia 1
Conidiobolus khandalensis (Balazy 1993, Nie et al. 2020)	Organic detritus 0		Globose to subglobose 17-21x14-18 0	Microconidia 1
**_Conidiobolus lamprauges_** (Balazy 1966, Nie et al. 2020)	Organic detritus & flies infected with _E. muscae_ 0		Globose to ovoid 13-20x15-22 0	Unknown 3
Conidiobolus lichenicola (Srinivasan & Thiralachar 1968)	Decomposing lichens 1		Pyriform 30x35 1	-//- 1
Conidiobolus macrosporus (Balazy 1993)	Organic detritus & living fungus		Globose to pyriform 25-	Microconidia 1

	gnats, narrow range 3	54x30-50 3	
Conidiobolus megalotocus (ibid.)	Organic detritus 0	Globose 12-55x10-48 1	-//- 1
Conidiobolus mycophagus (Srinivasan & Thiralachar 1965)	Decomposing fungi 1	Globose 25-33x35-40 1	Microconidia or like globose primaries 1
Conidiobolus mycophilus (Balazy 1993, Index Fungorum)	Decomposing fungi 1	Subglobose 25-40x20-33 1	Microconidia 1
Conidiobolus obscurus (Balazy 1993, ARSEF)	Living aphids, narrow range, weak 3	Globose 29-34 1	Like primaries 1
Conidiobolus polyspermus (Drechsler 1961, Index Fungorum)	Organic detritus 0	Globose 12-48x15-55 1	Unknown 3
Conidiobolus polytocus (Drechsler 1955c)	Organic detritus 0	Subspherical 14-29x12-25 1	Microconidia 1
Neoconidiobolus couchii = Conidiobolus couchii (Balazy 1993)	Organic detritus 0	Ovoid 13-27x10-16 2	Like primaries or elongate 2
Neoconidiobolus lachnodes = Conidiobolus lachnodes (ibid.)	Organic detritus & forest litter 3	Globose 10-27x8-25 0	Like primaries 1
Neoconidiobolus mirabilis = Conidiobolus mirabilis (Nie et al. 2018, Index Fungorum)	Organic detritus 0	Subglobose 21-28x17-21 1	-//- 1
Neoconidiobolus osmodes = *Conidiobolus osmodes* (Balazy 1993, ARSEF)	Organic detritus & dead insects & living insects, wide range 2	Globose to ovoid 25-37x22-30 2	-//- 1
Neoconidiobolus pachyzygosporus = Conidiobolus pachyzygosporus (Nie et al. 2018, Index Fungorum)	Organic detritus 0	Globose 16-23x11-18 0	-//- 1
Neoconidiobolus sinensis = Conidiobolus sinensis (Nie et al. 2012)	Organic detritus 0	Globose to pyriform 18-25x23-33 2	-//- 1
Neoconidiobolus stilbeus = Conidiobolus stilbeus (Nie et al. 2016, Index Fungorum)	Organic detritus 0	Globose 21-25x17-21 1	-//- 1
Neoconidiobolus stromoideus = Conidiobolus stromoideus (Balazy 1993)	Organic detritus & fly infected with *E. muscae* 0	Globose to pyriform 21-39x18-35 2	-//- 1
Neoconidiobolus thromboides = *Conidiobolus thromboides* (ibid.)	Organic detritus, fungi & dead & living insects, wide range 2	Globose 24-32x18-27 1	-//- 1
Neoconidiobolus vermicola = Macrobiotophthora vermicola (McCulloch 1977, Balazy 1993, Nie et al. 2020)	Nematodes 3	Obovoid 16-22x9-14 2	Secondaries occur but not described 2
Microconidiobolus nodosus = Conidiobolus nodosus (Index Fungorum)	Organic detritus 0	Globose 13-16x17-22 0	-//- 1
Microconidiobolus paulus = *Conidiobolus paulus* (Balazy 1993, ARSEF) *C. undulatus* is a synonym of this species	Organic detritus 0	Globose or ovoid 5-10x4-17 0	Like primaries 1
Microconidiobolus terrestris = Conidiobolus terrestris (Srinivasan & Thiralachar 1968, Nie et al. 2020, Index Fungorum)	Organic detritus 0	Globose 8-12 0	-//- 0

Batkoa apiculata (Balazy 1993)	Living insects 2	Globose 29-34x28-35 1	-//- 1
Batkoa gigantea (ibid.)	-//- 2	Globose 92x77 1	-//- 1
Batkoa major (ibid.)	-//- 2	Spherical to pyriform 48-52x41-47 1	-//- 1
Entomophaga aulicae (ibid.)	-//- 2	Pyriform and obovoid 29-38x20-30 2	-//- 2
Entomophaga conglomerata (ibid.) = *Batkoa apiculata*	-//- 2	Broadly pear-shaped 40-48x29-35 2	-//- 2
Entomophaga maimaga (ibid.)	-//- 2	Pyriform 26x21 2	-//- 2
Entomophthora chromaphidis (Humber and Feng 1991, Keller 2002)	-//- 2	Campanulate 12-16x16-14 1	-//- 2
Entomophthora culicis (Balazy 1993)	-//- 2	Campanulate 11-16x7-12 1	-//- 2
Entomophthora grandis (Keller 2002)	-//- 2	Campanulate 10-32x24-26 1	-//- 2
Entomophthora muscae (Balazy 1993)	-//- 2	Campanulate 20-32x15-28 1	-//- 2
Entomophthora planchoniana (ibid.)	-//- 2	Campanulate 15-20x13-17 1	-//- 2
Entomophthora scatophagae (Steinkraus & Kramer 1988)	-//- 2	Campanulate 29x23 1	-//- 2
Entomophthora schizophorae (Balazy 1993)	-//- 2	Campanulate 17-25x12-22 1	-//- 2
Entomophthora syrphi (Balazy 1993, Keller 2002)	-//- 2	Campanulate 28-32x21-27 1	-//- 2
Entomophthora thripidi (Balazy 1993)	-//- 2	Campanulate 10-15x8-12 1	-//- 2
Erynia conica (ibid.)	-//- 2	Fusiform to conical 27-80x12-14 2	Like primaries or tetraradiate 2
Erynia ovispora (ibid.)	-//- 2	Ovoid or ellipsoid 23-29x12-14 2	Like primaries 2
Erynia rhizospora (Thaxter 1988, Balazy 1993)	-//- 2	Oblong clavate to crescent-shaped 30-35x8-10 2	Like primaries or spherical 2
Erynia sciarae (Balazy 1993)	-//- 2	Ovoid to turbinate 18-25x12-16 2	Unknown 3

Eryniopsis caroliniana (ibid.)	-//- 2	Oblong ovoid or cylindrical 22-51x11-19 2	Like ovoid primaries 2
Furia americana (ibid.)	-//- 2	Oblong ovoid 28-30x14 2	Like primaries 2
Furia gastropachae (ibid., Filotas et al. 2003)	-//- 2	Oblong ovoid 17-25x7-12 2	More globose than primaries 2
Furia ithacensis (Balazy 1993)	-//- 2	Narrow ovoid to ellipsoid 15-24x9-12 2	More ovoid than primaries 2
Furia neopyralidarum (ibid.)	-//- 2	Pyriform or ovoid 16-28x12-17 2	Like primaries 2
Furia pieris (ibid.)	-//- 2	Ovoid 21-32x12-24 2	Like primaries but more ovoid 2
Furia virescens (ibid.)	-//- 2	Elongate cylindric or claviform 28-29x10-11 2	Shorter and broader than primaries with convex papilla 2
Massospora cicadina (Soper 1974)	-//- 2	Ovoid & verrucose 10-17x14-20 2	Absent 3
Massospora platypediea (Macias et al. 2020)	-//- 2	Ovoid & verrucose 11-18x15-20 2	Absent 3
Pandora blunckii (Balazy 1993)	-//- 2	Pyriform or ovoid 15-18x7-9 2	Like primaries 2
Pandora delphacis (ibid.)	-//- 2	Ovoid to ellipsoidal 29-36x13-18 2	Ovoid 2
Pandora dipterigena (ibid.)	-//- 2	Elongate, variable shape 22-30x11-15 2	Ovoid to somewhat pear-shaped 2
Pandora kondoiensis (ibid.)	-//- 2	Narrow ellipsoid 16-21x10-12 2	Ovoid or short pyriform 2
Pandora neoaphidis (ibid.)	-//- 2	Ovoid or ellipsoid 21-32x11-14 2	Like primaries but more variable 2
Zoophthora anglica (ibid.)	-//- 2	Cylindric 23-30x9-10 2	Capilliconidia or like primaries 2
Zoophthora lanceolata (ibid.)	-//- 2	Ellipsoid or cylindrical 15-21x5-7 2	-//- 2
Zoophthora phalloides (ibid.)	-//- 2	Cylindrical 23-25x7-8 2	Capilliconidia or like primaries but shorter 2

Zoophthora radicans (Balazy 1993, Keller 1991)	-//- 2	Subcylindrical to subfusiform 20-23x6 2	Capilliconidia or like primaries 2
Strongwellsea castrans (Balazy 1993, Eilenberg et al. 2020)	-//- 2	Elongate ovoid 25-33x12-19 2	Ellipsoid or subglobose 2
Outgroup taxa			
Coemansia reversa (Young 1973)	Organic detritus 0	Non-forcible ovoid sporangiospore 4x2 3	Absent 0
Schizangiella serpentis (Dwyer et al. 2006)	Animals 3	Unknown 3	Unknown 3
Smittium culisetae (Wang et al. 2013)	Commensal of living insects 2	Non-forcible trichospore 16x4 3	Absent 0
Syncephalis pseudoplumgaleata (Lazarus et al. 2017)	Contaminant of fungi 1	Non-forcible variously shaped merospores 3	Absent 0

-//- – same as above.

Lifestyle: non-pathogenic to insects, grow well on artificial media (0), insect pathogen-generalist, grow well on artificial media (1), insect pathogen of wide range (2), insect pathogen of narrow range (3).

Primary conidia: forcible up to and equal to 20 μm in diameter – small (0), forcible >20 in diameter – large (1), forcible other than round (2), not forcible or unknown (3).

Secondary conidia: absent (0), microconidia (1), presence of capilliconidia or other shape than globose (2), unknown (3).

In bold – genomes or transcriptomes available, underlined – taxa used in Nie et al. phylogeny (2020).

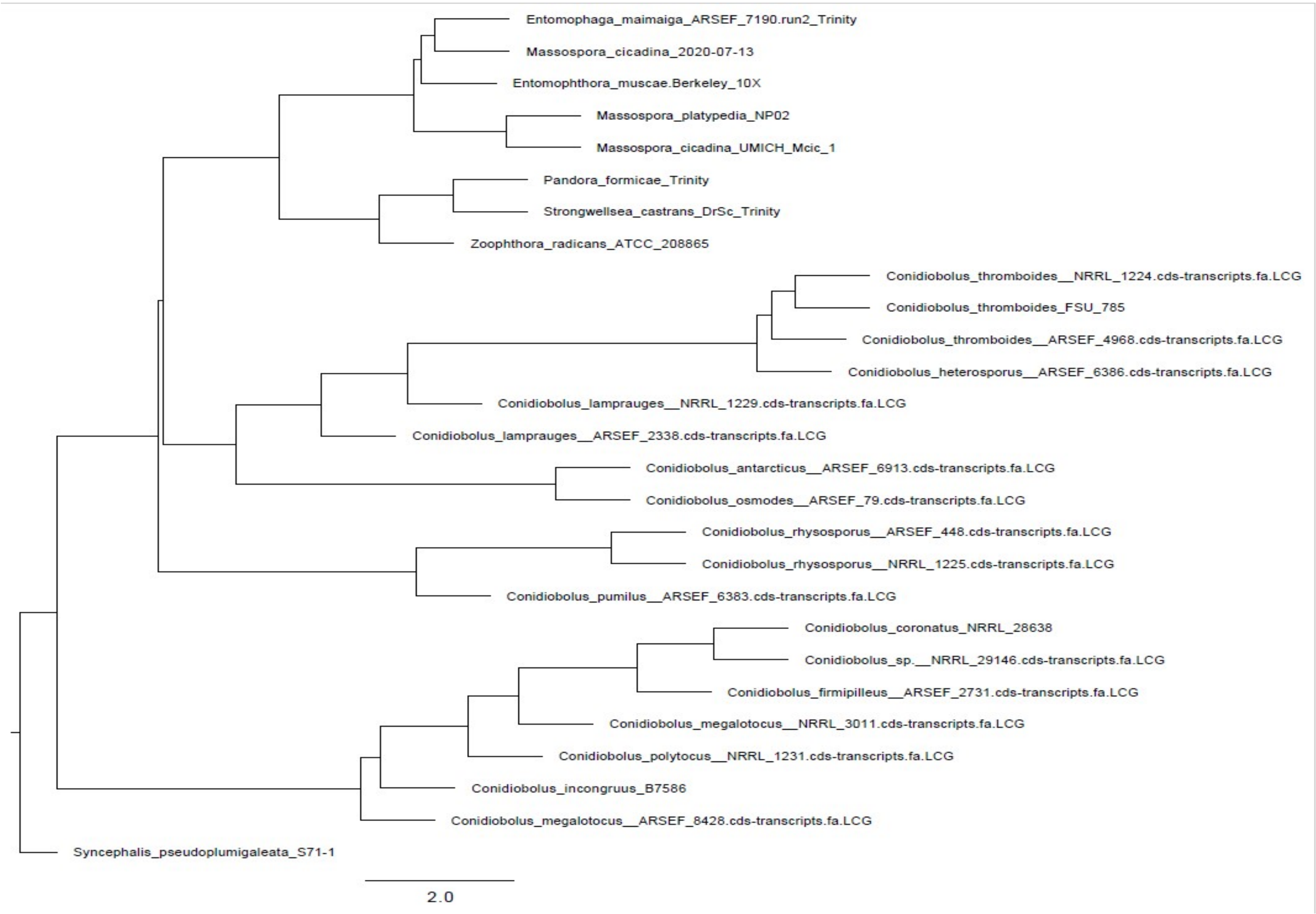

Supplemental Figure S1. Phylogeny based on 27 genomes of *Conidiobolus*-like fungi. Major clades correspond to the families *Conidiobolaceae*, *Neoconidiobolaceae* and *Capillidiuaceae* (from the bottom up). Outgroup *Syncephalis pseudoplumigaleata* S71-1.

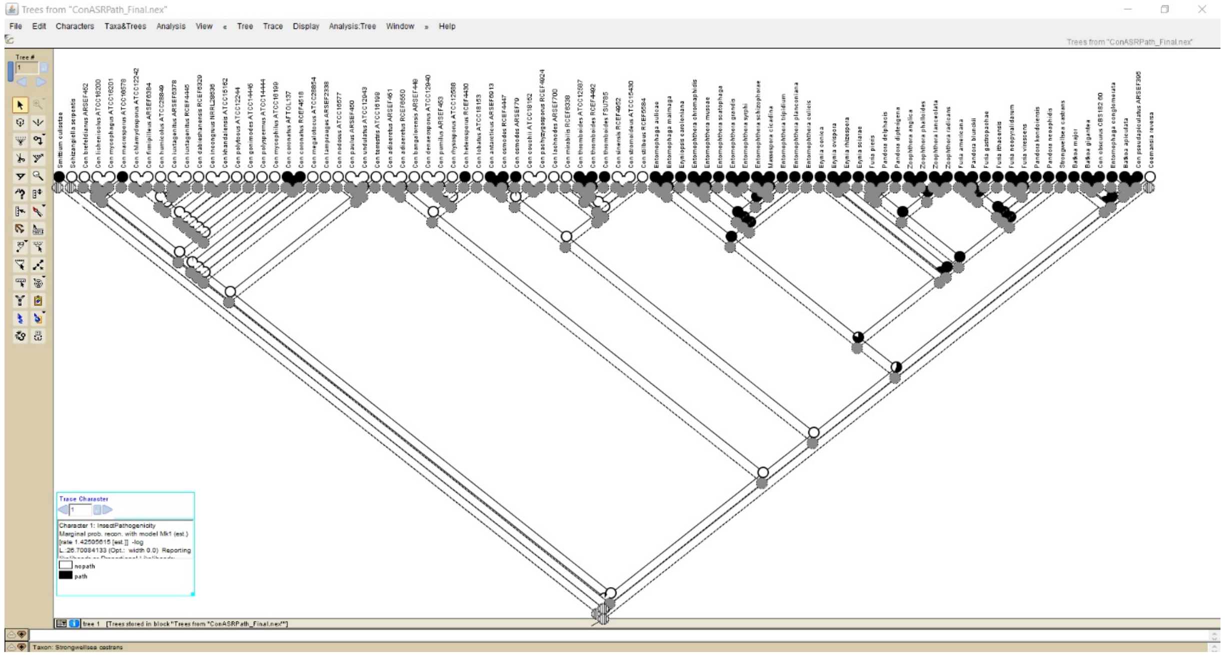

Supplemental Figure S2. Ancestral state reconstruction of insect-pathogenic lifestyle and ballistic conidia (gray), which evolutionary precedes parasitic lifestyle (black) for two states: present (black) and absent (white).
